# Evidence of a largely staminal origin for the *Jaltomata calliantha* (Solanaceae) floral corona

**DOI:** 10.1186/s13227-019-0122-9

**Published:** 2019-04-19

**Authors:** Jamie L. Kostyun, Josephine E. Robertson, Jill C. Preston

**Affiliations:** Department of Plant Biology, The University of Vermont, 111 Jeffords Hall, 63 Carrigan Drive, Burlington, VT 05405 USA

**Keywords:** ABCDE genes, Corona, *Jaltomata*, Nectar, Novel floral organs

## Abstract

**Background:**

Understanding the evolution of novel features requires homology assessments at different levels of biological organization. In flowering plants, floral coronas that play various roles in plant–pollinator interactions have evolved multiple times independently, but are highly variable in their final position and overall morphology. Coronas of the Solanaceae species *Jaltomata calliantha* are found between the corolla and stamens, adjacent to the gynoecium, and form cups that house copious amounts of their characteristic blood red nectar. To test the hypothesis that *J. calliantha* coronas evolved as an outgrowth of stamens and therefore have staminal identity, we assessed their development, floral organ identity gene expression, and cellular morphology.

**Results:**

*Jaltomata calliantha* coronas emerge after the initiation of all conventional floral organs on the abaxial side of the proximally modified stamens and then expand medially and laterally to form nectar cups. Overlapping expression of the B-class organ identity genes *JcAPETALA3* and both *JcPISTILLATA/GLOBOSA* orthologs (*JcGLO1* and *JcGLO2*), and the C-class-like gene *JcAGAMOUS1*-like, unites the stamens and corona. Epidermal cell shape also connects the adaxial surface of coronas and petals, and the stamen base, with remaining floral organs showing divergent cell types.

**Conclusions:**

Our data, based on multiple lines of evidence, support a largely staminal origin for *J. calliantha* coronas. However, since slightly enlarged stamen bases are found in *Jaltomata* species that lack coronas, and *J. calliantha* stamen bases share cell types with petals, we hypothesize that stamen bases recruited part of the petal identity program prior to fully expanding into a corona.

**Electronic supplementary material:**

The online version of this article (10.1186/s13227-019-0122-9) contains supplementary material, which is available to authorized users.

## Background

The evolution of novel structures—such as leaves, limbs, and flowers—has occurred countless times across the tree of life [1–3]. However, while such structures appear novel at the morphological and/or functional level, they often emerge from the rewiring or redeployment of ancient developmental genetic pathways, resulting in complex homology relationships at different levels of organization [4, 5]. Oral teeth of jawed vertebrates, for example, are known to be shaped by a deeply conserved gene regulatory network (GRN) that is shared with skin denticles. Despite this, teeth emerge from epithelial cells associated with taste bud development, suggesting that the latter uniquely coopted the denticle GRN [6]. Such a hierarchical assessment of homology [4], taking multiple lines of evidence into account, is the key to understanding the evolution of novelty and ultimately of how the diversity of life originated.

In many angiosperms, several such morphological novelties are found within flowers that have ambiguous relationships to conventional floral organs, including crown-like outgrowths often referred to as coronas. In daffodils (*Narcissus* sp., Amaryllidaceae), the corona arises between the stamen and the inner tepal whorl and superficially resembles petals in terms of shape and pigmentation. Despite this, expression of floral organ identity MADS-box genes suggests a strong affinity between coronas and stamens, whereas developmental analyses suggest positional origins from the underlying hypanthium [7]. Coronas have also been described from several other, distantly related taxa [8, 9]. This includes the remarkable diversity of coronas in the genus *Passiflora* (passionflowers; Passifloraceae), which range in form from small, inconspicuous structures to organs having stunningly complex combinations of colors, patterns, and appendages [10], and those that are derived within the Solanaceae genus *Jaltomata*. Despite their independent origins, MADS-box gene expression in most organs of *Passiflora* coronas is similar to that in daffodil coronas, again suggesting cooption of a stamen identity program into this novel structure [7, 10]. The extent to which this molecular cooption can be generalized to other coronas that are developmentally and morphologically distinct, including those within *Jaltomata*, is still a matter of debate.

The use of MADS-box gene expression as one source of data with which to evaluate homology is based on the largely conserved ABC model of floral organ identity specification (but see [11] for divergence of A-class function), whereby the A-class genes *APETALA1/SQUAMOSA* (*AP1/SQUA*) and *APETALA2/LIPLESS1/2* (*AP2/LIP1/LIP2*) specify sepal identity, A- and the B-class genes *APETALA3/DEFICIENS* (*euAP3/DEF*) and *PISTILLATA/GLOBOSA* (*PI/GLO*) specify petal identity, B- and the C-class gene *AGAMOUS/PLENA* (*AG/PLE*) specify stamen identity, and the C-class gene alone specifies carpel identity [12]. Of these five genes, all but *AP2* are members of the MADS-box family of transcription factors. Although the initial ABC model was defined based on work in *Arabidopsis thaliana* (Brassicaceae) and *Antirrhinum majus* (Plantaginaceae), similar expression patterns and functions have been assigned to most homeotic gene orthologs of the Solanaceae species petunia (*Petunia* × *hybrida*) and tomato (*Solanum lycopersicum*) (reviewed in [13, 14]). One exception is the *AP1/SQUA* ortholog that has been lost from the petunia genome [15], although the tomato *AP1/SQUA*-like gene *MACROCALYX* (*MC*) has been found to affect sepal size, but not identity [16]. The petunia *AP2/LIP1/LIP2*-like gene *PhAP2A* is expressed most strongly as expected in the first and second whorls, whereas the *euAP3/DEF*-like gene *PhDEF* (also known as *GREEN PETAL* [*GP*] and *PMADS1*) and *PI/GLO*-like genes *PhGLO1* (also known as (*FLORAL BINDING PROTEIN* [*FBP1*]) and *PhGLO2* (also known as *PMADS2* and *FBP3*) are expressed most strongly in the second and third whorls where they affect the identify of petals and stamens [17]. The duplicated *AG*-like genes *pMADS3* and *FBP6* are similarly expressed in a conserved manner within the third and fourth whorls [14, 18]. Taken together, these and further data from tomato [19, 20] predict that expression and function of the B-, C-, and at least the *AP2*-like A-class floral homeotic gene orthologs will be conserved in *Jaltomata.*

In addition to the A, B, and C floral homeotic genes, two additional classes of MADS-box transcription factors have been implicated in floral organ identity. The D-class genes *SEEDSTICK (STK)* in *A. thaliana*, *TAGL11* in tomato [21], and *FBP7/FBP11* in petunia are all expressed strongly in ovaries, with mutations in these genes causing ovary to style/stigma homeotic transformations in at least *A. thaliana* and petunia [22, 23]. In tomato, *TAGL11* is additionally expressed in the tapetum of anthers [21], but the functional significance of this has yet to be determined. In contrast to D-class genes, E-class gene products in eudicots largely stabilize homo- and heterodimers of A, B, C, and D proteins, thus forming organ-specific quartets that regulate the downstream transcription of genes involved in floral organ patterning (reviewed in [24]). In *A. thaliana*, three out of the four E-class genes (*SEPALLATA1, SEP2, SEP3*, and *SEP4*) need to be mutated in order to see the strong homeotic phenotype of all floral organs converted to sepals [25, 26], whereas cosuppression of petunia E-class genes *FBP2* and *FBP5* in petals, stamens, and carpels causes a similar phenotype [27].

*Jaltomata* coronas are novel and have been described only in the monophyletic *Modillonia* section, comprised of sister species *J. calliantha, J. quipuscoae,* and *J. aspera* [28, 29]. The lack of a corona in the rest of *Jaltomata*, as well as the *Solanum* outgroup, indicates that it is a derived trait that evolved only once in the genus [29]. Unlike the previously described coronas of daffodils and *Passiflora,* coronas of campanulate *J. calliantha* flowers, as well as those of the other species within the *Modillonia,* take the form of cups that fill with vivid red nectar that appears to act as an attractant and reward for hummingbird pollinators [30, 31]. A bowl of tissue forms between each of the five stamens, producing five cups that each appear to radiate from the base of the filament and surround the superior gynoecium [32]. Based on this late-stage positional information, the *Jaltomata* corona might best be interpreted as an outgrowth of stamens, or as stemming as unique primordia from the underlying receptacle.

In this study, we test the hypothesis that derived *J. calliantha* coronas evolved from recruitment of the stamen organ identity protein complex (PI/GLO, AP3/DEF, AG/PLE, and SEP3/FBP2/FBP5-like) and assess the developmental and cellular origins of these novel structures. We find that positional developmental information, cell micromorphology, and patterns of gene expression support a predominantly staminal identity of coronas, consistent with outgrowth of the corona tissues from the abaxial margins of stamen bases.

## Methods

### Plant growth and tissue collection

Several *J. calliantha* plants were grown to reproductive phase in an 18–21 °C controlled greenhouse at the University of Vermont under 14-h light conditions with weekly fertilization. Three to five biological replicates of leaves, whole flower buds, and dissected floral tissues were then collected in RNA later (Thermo Fisher Scientific, Waltham, MA, USA), fixed in FAA (3.7% formaldehyde, 50% alcohol, 5% glacial acetic acid), or snap-frozen in liquid nitrogen for downstream analysis.

### Microscopy

Fresh flowers of *J. calliantha* at different developmental stages were imaged using a Leica MZ8 stereoscope. To increase the resolution of cell morphology, FAA-fixed tissues were taken through an alcohol series to 100% ethanol at room temperature, and either critical point dried, sputter-coated with argon, and imaged with a JEOL6060 scanning electron microscope, or stained with 1% Toluidine blue and imaged using a Leica LED microscope.

### RNA extraction and cDNA synthesis

RNA was extracted from each tissue sample using TRI Reagent (Thermo Fisher Scientific, Waltham, MA), and contaminating DNA was removed using the TURBO DNase kit (Thermo Fisher Scientific, Waltham, MA, USA), both according to the manufacturer’s instructions. Samples included leaves, dissected sepals, petals, corona, stamens, and gynoecium from mature flowers, as well as dissected sepals, petals, stamens, and gynoecium from pre-corona emergence buds. RNA concentration was estimated using a Quantus Fluorometer (Promega), and 500 ng of each sample was added to iScript cDNA Synthesis kit (BIO-RAD, Portland, ME, USA) half (10 µl) reactions to generate cDNA. Each 10-µl reaction was then diluted 1:10 in water to produce a working solution for PCR.

### Phylogenetic analysis

We used a previously assembled and annotated *J. calliantha* transcriptome dataset [29], consisting of RNA from both vegetative and reproductive stages of growth, to find putative MADS-box genes belonging to the *AP1/FUL* (A-class), *PI/AP3* (B-class), *AG* (C-class), *AGL11* (D-class), and *SEPALLATA* (*SEP*) (E-class) clades [24, 33]. Nucleotide sequences for each putative homeotic gene homolog were then aligned with A-, B-, C-, D-, and E-class MIKC MADS-box genes downloaded from Phytozome 12 (http://phytozome.jgi.doe.gov/pz/portal/html) or the Sol Genomics Network (https://solgenomics.net) from *A. thaliana*, petunia, and *Aquilegia* sp. using MAFFT [34], with fine-tuning by eye in Mesquite v3.5.1 [35]. The final alignment was submitted to MrBayes v3.2.6 in XSEDE through the CIPRES portal v3.3 for Bayesian phylogenetic analysis [36, 37], using default parameters, except that each of the two runs comprised 10 million iterations. The majority-rule consensus tree was visualized in FigTree v1.4.3 (tree.bio.ed.ac.uk/software/figtree) using the *AP1/FUL* clade as an outgroup.

### Quantitative (q) PCR

Primers were designed for representative A-, B-, C-, and E-class gene orthologs, and the two housekeeping genes *EF1alpha* and *UBQ5*, using Primer3 version 4.1.0 [38] (Table 1). Primer pair efficiencies were determined using a dilution series of pooled *J. calliantha* cDNAs in 20 µl iTaq Universal SYBR Green (BIO-RAD, Portland, ME, USA) reactions. Each reaction was run in triplicate on a StepOne real-time PCR machine (Thermo Fisher Scientific, Waltham, MA, USA) with 60 °C annealing and otherwise recommended conditions. Only primer pairs amplifying products with single melt curves and efficiencies between 90 and 110% (Table 1) were used for quantification of gene expression across different replicated *J. calliantha* tissues according to the delta cT method. cT values were averaged across technical triplicates and corrected for both primer efficiencies and the geomean of the two housekeeping gene values.

**Table 1:** Primers used in this study

Forward primer (5′–3′)	Reverse primer (5′–3′)	Efficiency (%)
*JcEF1a*.A.F: AGGCTGGTGGTGATTAGATGA	*JcEF1a*.A.R: AGCTGCAAACATAATCCCATAATT	102
*JcUBQ5.*A.F: TTCGCTGAGTACCCACCATT	*JcUBQ5.*A.R: CCTGCACTGTTCACTTTCCC	92
*JcTAG*.F4: GAGAGCTCAGCATCATCAGC	*JcTAG*.R4: GGGTTGGTCTTGTCTAGGGT	100
*JcTAGL1*.19Fa: AATCCCCATTACTCTCGCCG	*JcTAGL1*.127Ra: GACCCTTGACCCAAATTTCAGA	96
*JcTM29*.15.F.b: ACAACCTGCAACAACCATGG	*JcTM29*.236.R.b: TCCGAGGGACATTGATTCACA	95
*JcTM6*.25.F.a: GGTGTAGTGGAAAATGAGGGG	*JcTM6*.174.R.a: TCTTCAGGACAGGCGTAGATC	101
*JcAP1*.F1: ATTGCAGCAAGGTGAATGGC	*JcAP1*.R1: CGTCGTGATAGTGAGCTCCT	103
*JcGLO1*.F1: ACCTCATTCTGTTTGTCACGG	*JcGLO1*.R1: ACTGGCAGAAGATTGTGGGA	95
*JcGLO2*.F2: GGAGAAGGCTATGGGATGCT	*JcGLO2*.R2: AAGATCTCAGACTGCTTGGC	100
*JcAP3*.F1: ACATTTGGCAACCCTTTCCA	*JcAP3*.R1: TCTTCCCACGAGCCATAACT	97

## Results

### Positional information supports a largely staminal origin for the *J. calliantha* corona

**Fig. 1:**
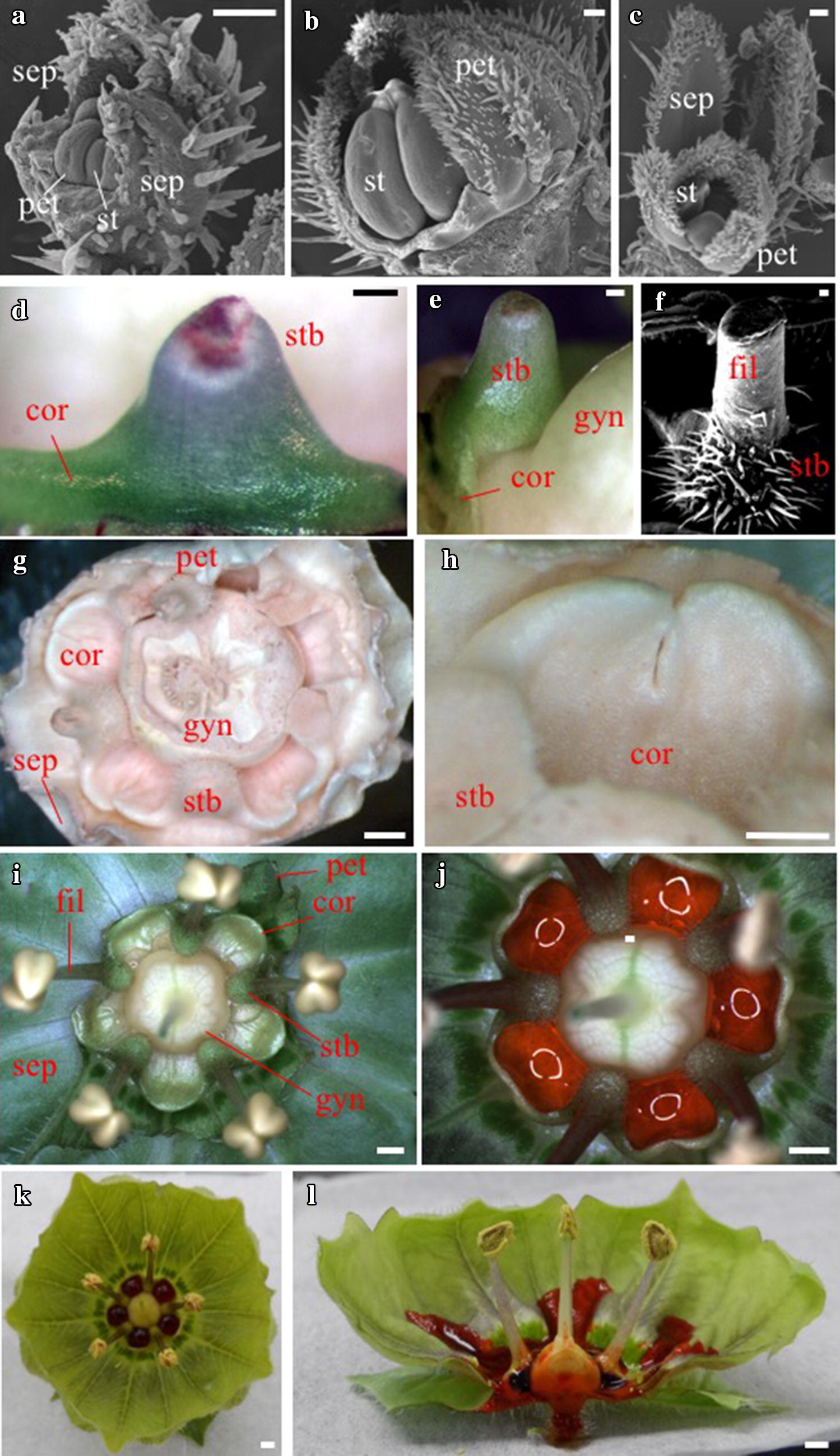
Emergence of the *J. calliantha* corona in late flower development. **a**–**c** Scanning electron micrographs of mid-stage flower buds, prior to a coronal outgrowth at the base of stamens. **d** Young stamen base with filament detached showing outgrowth of the corona. **e** Side view of **d** indicating outgrowth of the corona on the abaxial side of the stamen around the base of the gynoecium. **f** Scanning electron micrograph of a fully expanded and mature stamen base covered with trichomes. **g** Dehydrated mature flower with stamen filaments and most of the perianth removed to reveal the corona lobes and swollen stamen bases. **h** Close-up of the two-lobed corona between two stamen bases from **g**. **i** Close-up of the inner organs of open flowers showing five stamens emerging from green swollen bases that contact the gynoecium base on their adaxial side. Petals have been trimmed back to reveal five corona cups between the swollen stamen bases, on top of the petal bases, and adjacent to the gynoecium. Secretion of light-colored nectar into the corona cups begins shortly after flower anthesis. **j**–**l** Nectar becomes darker red as the flower ages, and pools in the slightly heart-shaped corona cups. *sep* sepal, *pet* petal, *st* stamen, *stb* stamen base, *fil* stamen filament, *cor* corona, *gyn* gynoecium. Scale bars = 100 µm for (**a**–**f**) and 1 mm for (**g**–**l**)

Conventional *J. calliantha* floral organs (i.e., sepals, petals, stamens, and gynoecia) initiated in four concentric whorls [32] (Fig. 1a–c), with the corona emerging later in development, following the differentiation of the stamen filaments and anthers (Fig. 1d, e). Corona tissues were first visible as a ridge on the abaxial side of stamens (Fig. 1d) that gradually extended medially across the inter-staminal space (Fig. 1e) to hug the base of the gynoecium and then laterally to fill the emerging gap between the stamens and gynoecium (Fig. 1g–i). The origin of individual corona initials from the abaxial base of each of the five stamens was evidenced by the two-lobed nature of each corona cup (Fig. 1h) at mid-stages of development. However, by late-stage growth, elongation of the corona cup edges was dominated by growth of a proximal ring primordium that emerged from the inter-staminal region, giving rise to five apparently single-lobed structures (Fig. 1i). Simultaneous with extension of the corona was the swelling of stamen bases that later developed numerous long trichomes (Fig. 1d–f). Similar to the corona, the extent of stamen base swelling is unique to section *Modillonia*, with other members of the genus having either laminar stamen filaments (e.g., *J. repandidentata*) (Additional file 1: Fig. S1a) or filaments that have only slightly expanded bases (e.g., *J. sinuosa*) (Additional file 1: Fig. S1c). Although the function of stamen base swelling is unknown, it may diminish leakage of nectar from the corona cups (Fig. 1j–l), especially as mature flowers are typically pendant among branches.

### Partial transformation of the stamen base provides a link between petal identity and corona cellular micromorphology

**Fig. 2:**
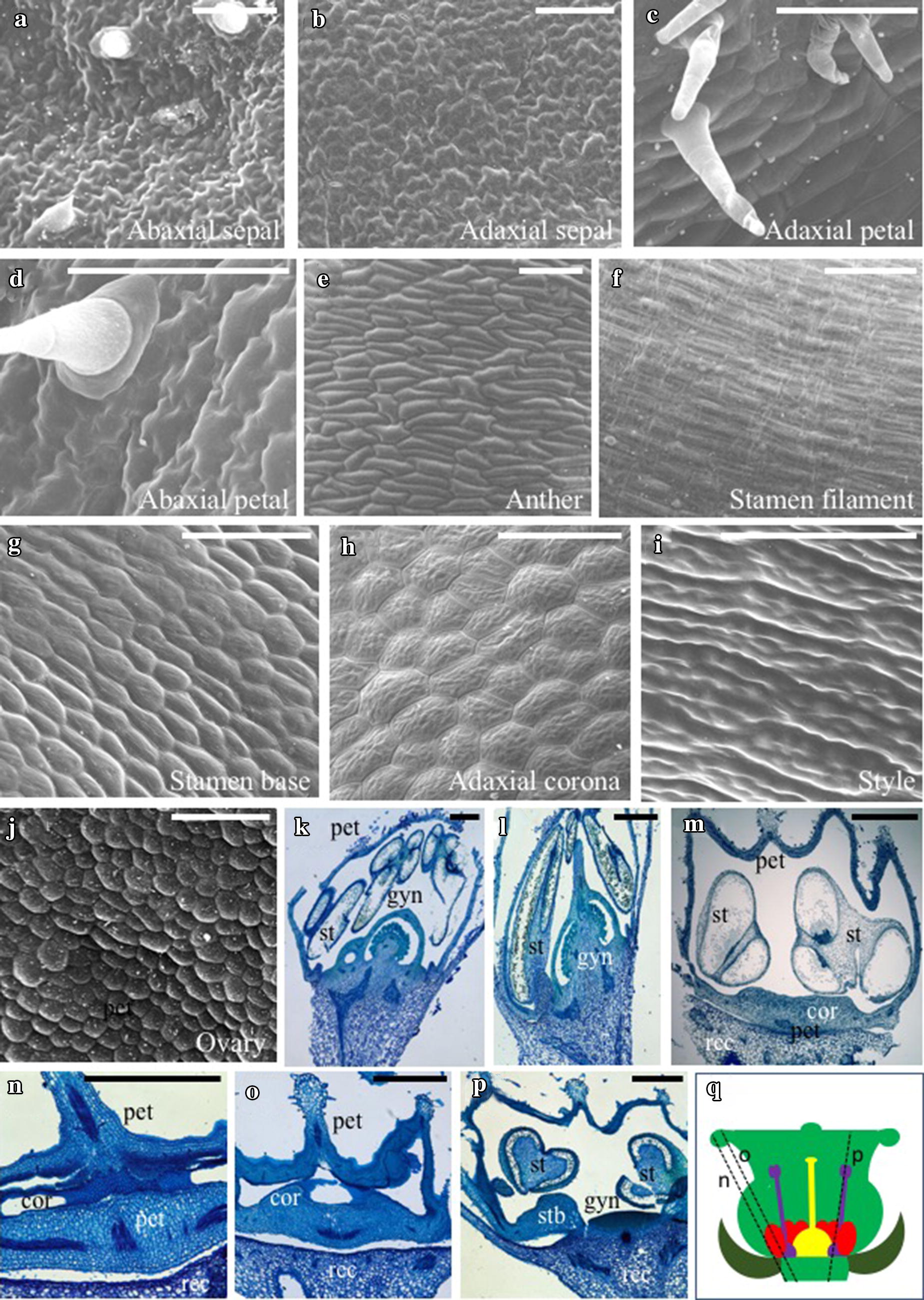
Cell morphologies of *J. calliantha* floral organs. **a** Jigsaw-shaped cells on the abaxial (lower) surface of the sepal. **b** Jigsaw-shaped cells on the adaxial (upper) sepal surface. **c** Slightly raised rounded cells of the adaxial petals. **d** Jigsaw-shaped cell of the abaxial petals. **e** Elongated cells of anthers. **f** Flattened and highly elongated cells of the stamen filaments. **g** Slightly raised rounded cells of the stamen base. **h** Slightly raised rounded cells of the corona. **i** Toothed elongated cells of the style. **j** Highly rounded cells of the ovary surface. **k**, **l** Sections through a mid-stage toluidine blue-stained flower bud showing mature conventional floral organs, prior to corona growth. **m** Initiation of corona development as a ring of tissue connecting the stamen bases. **n**, **o** Late-stage corona and petal development revealing similar inner layers of dark blue-stained rapidly dividing cells. **p** Section from the same flower as **k**, **l** toward the center of the flower showing the swollen stamen base stamen thecae. **q** Cartoon of a wax-embedded *J. calliantha* flower illustrating the angle of sectioning in **n**–**p**. Note that the fused corolla (light green) is artificially pushed upward to enclose the inner corona (red), stamens (purple), and gynoecium (yellow). *pet* petals, *st* stamens, *gyn* gynoecium, *rec* receptacle, *cor* corona, *stb* swollen stamen base. Scale bars = 100 µm for (**a**–**j**) and 1 mm for (**k**–**p**)

Examination of individual cells within the emerging corona (Fig. 2h) showed similarities with the rounded and raised epidermal cells at the base of the stamens (Fig. 2g) and adaxial petal surface (Fig. 2c), but lacked the trichomes found on both these structures. In contrast, cells on both sides of the sepal (Fig. 2a, b) and abaxial petal (Fig. 2d) were jigsaw-shaped, whereas cells of the anther (Fig. 2e), stamen filament proper (Fig. 2f), and style (Fig. 2i) were flattened and elongated, and those on the ovary surface were small and very round (Fig. 2j). The difference in cell types between the proximal (i.e., stamen base) and distal regions of the stamen filaments is consistent with partial transformation of stamen base identity toward a more petal-like micromorphology, which can then be extended into the stamen–stamen and stamen–carpel boundary regions to form the corona. Supporting this, similarly sized, shaped, and toluidine blue-stained cells were found on outer surface sections of petals, the corona, and stamen bases (Fig. 2k–m). Furthermore, small cells associated with the inner layer of petals and the corona appeared to be rapidly dividing and deeply stained blue (Fig. 2n, o); these cells were not apparent in any sections through the stamens or gynoecia (Fig. 2n–p).

### MADS-box gene retention and expression is largely consistent with a conserved ABCDE model in *J. calliantha*

**Fig. 3:**
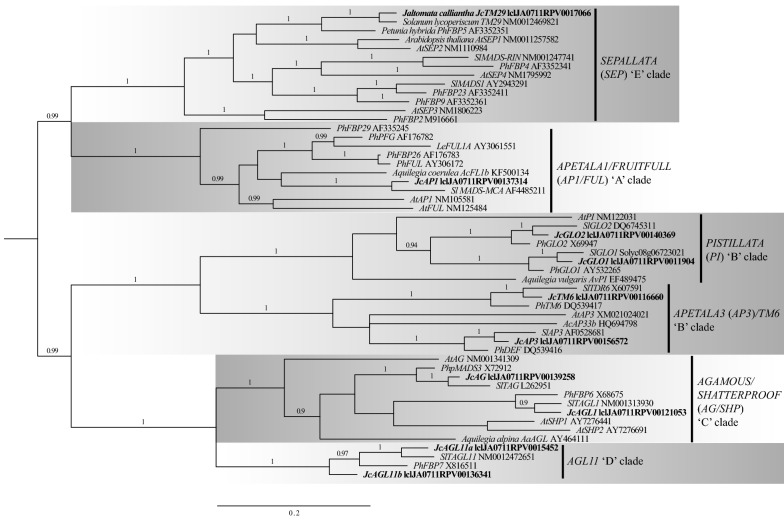
Bayesian majority-rule tree of angiosperm (*A. thaliana*, Solanaceae, and *Aquilegia* sp.) MIKC MADS-box genes from the ABCDE clades. *J. calliantha* genes are highlighted in bold. Posterior probability support values are shown above each branch if > 0.90

A Bayesian phylogenetic analysis of putative *J. calliantha* orthologs to the A-, B-, C-, D-, and E-class MADS-box genes from *A. thaliana*, petunia, tomato, and the basal eudicot *Aquilegia* sp. (Ranunculaceae) revealed six strongly supported (posterior probability > 0.9) monophyletic clades containing *AP1/SQUA*- (A-class), *AP3/DEF*- (B-class), *PI/GLO*- (B-class), *AG*- (C-class), *AGL11*- (D-class) and *SEP*-like (E-class) genes as expected based on [33] (Fig. 3). Single orthologs to the tomato *AP1*-like A-class gene (*LEMADS*-*MC*), and Solanaceae *AG*-like (tomato *TAG1* and petunia *PhpMADS3*) and *SHATTERPROOF1/2* (*SHP1/2*)-like (tomato *TAGL1*) C-class genes were found for *J. calliantha*. Similar to previous analyses, the majority-rule Bayesian phylogeny supported a duplication of *AP3/DEF*- and *PI/GLO*-like genes within or at the base of Solanaceae, with *J. calliantha* having an ortholog of each, i.e., *JcGLO1, JcGLO2, JcAP3*, and *JcTM6*. For the E-class *SEP*-like genes, one *J. calliantha* gene orthologous to tomato *TM29* and petunia *FBP5* was revealed, but no orthologs of tomato *MADS1*, *MADS*-*RIN*, or petunia *PhFBP2* were inferred, probably due to incomplete sampling. Finally, two *AGL11*/*PhFBP7*-like D-class genes (designated *JcAGL11*-*a* and *JcAGL11*-*b*) were found in the *J. calliantha* transcriptome (Fig. 3).

**Fig. 4:**
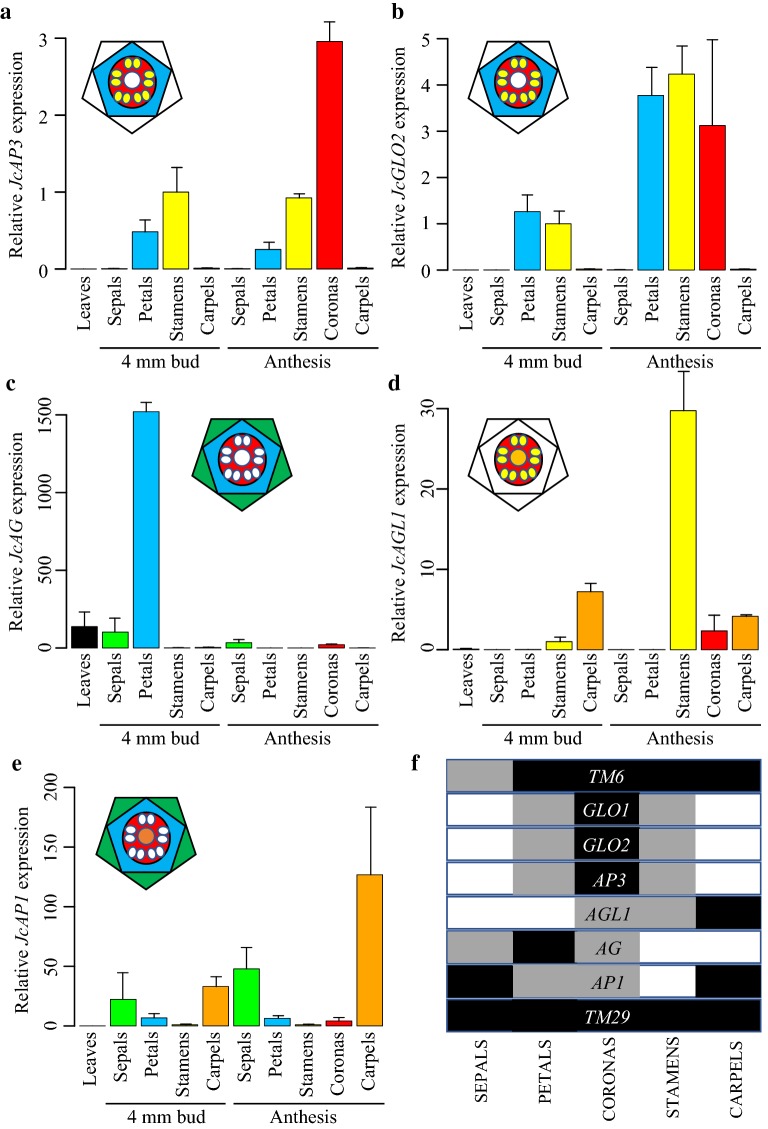
Quantitative RT-PCR of differentially expressed ABC genes across *J. calliantha* floral organs and development. **a** *JcAPETALA3* (*JcAP3*) is expressed as predicted in petals and stamens, and in late emerging coronas. **b** *JcPISTILLATA*/*JcGLOBOSA2* (*JcPI/JcGLO2*) is expressed as predicted in petals and stamens, and in late emerging coronas. **c** *JcAGAMOUS* (*JcAG*) is unexpectedly expressed in leaves, sepals, and petals, and in late emerging coronas. **d** *JcAGAMOUS*-*LIKE 1* (*JcAGL1*) is expressed as predicted in stamens and carpels, and in late emerging coronas. **e** *JcAPETALA1* (*JcAP1*) is expressed broadly in floral organs, but is not detected in stamens. **f** Overlapping expression domains in each floral organ. Black, strong relative expression; gray, weak relative expression. Bars in graphs denote averages of three biological replicates with standard errors. Colors mark expression in the same floral organs between the graphs and floral diagrams. Leaves, black; sepals, green; petals, blue; stamens, yellow; coronas, red; and carpels, orange

Relative expression analyses of the putative B-class genes *JcAP3*, *JcGLO1*, and *JcGLO2* demonstrated high expression levels in petals and stamens of both 4 mm young buds and post-anthesis flowers, with little to no transcripts detected in leaves, sepals, or carpels (Fig. 4a, b; Additional file 2: Fig. S2a). Thus, expression of *JcAP3* and both *JcGLO1* and *JcGLO2* was fully consistent with the ABCDE model. In contrast, the *JcAP3* paralog *JcTM6* was expressed in all conventional floral organs, as well as in leaves (Additional file 2: Fig. S2b). In the case of the putative A-class MADS-box gene *JcAP1*, expression was found in sepals, petals, and carpels, but not leaves or stamens (Fig. 4e). This expression pattern is broader than that found for A-class genes in *A. thaliana*; however, studies on several other angiosperm eudicots have found *AP1*-like expression in both the perianth and carpels (reviewed in [11]). Similar to the AP3/DEF clade genes, the two *J. calliantha* members of the AG clade showed divergent patterns of expression. Specifically, whereas the *SHP1/2*-like C-class gene *JcAGL1* fit the prediction of discrete expression within the stamens and carpels at different flower stages (Fig. 4d), the actual *AG* C-class gene ortholog *JcAG* was unexpectedly expressed in leaves and sepals, and very strongly in 4 mm bud petals (Fig. 4c). On closer inspection of the translated *JcAG* amino acid sequence, we found a unique 98 bp insertion in the K domain [39], 75 amino acids upstream of the conserved stop codon, inside which was a premature stop codon that might explain the non-predicted expression domain. Finally, the E-class *SEP1/2* ortholog *JcTM29* was expressed broadly across conventional organs as has been found for many *SEP*-like genes, but transcripts were not detected in leaves (Additional file 2: Fig. S2c).

### MADS-box gene expression further supports stamen identity for the corona

For genes that showed the predicted expression pattern in conventional organs according to the ABC model, all were expressed to some extent in coronas. Thus, *JcGLO1*, *JcGLO2*, and *JcAP3* expression linked corona identity with petals or stamens (Additional file 2: Fig. S2a; Fig. 4a, b), whereas *JcAGL1* expression linked corona identity with stamens or carpels (Fig. 4d). Using the floral quartet model as a framework to infer the functional consequences of gene expression, and assuming conservation of protein interactions in *J. calliantha*, the overlap between *JcGLO1, JcGLO2, JcAP3*, *JcAGL1*, and at least one *JcSEP*-like gene (*JcTM29*) (Additional file 2: Fig. S2c), strongly supports a staminal origin for coronas (Fig. 4f). On the other hand, the fact that *JcAGL1* and *JcAP1* were both expressed in coronas (Fig. 4d,e), but not stamens, might be important for modification of the stamen identity floral quartet, giving coronas a somewhat unique identity.

## Discussion

A key role for the field of evolutionary developmental biology is to reveal the underlying mechanisms for the evolution of novel features, i.e., phenotypes that emerge with ambiguous homology to traits present in sister lineages and their inferred common ancestor, and to determine the extent to which convergent phenotypes have been affected by modifications to the same tissues, developmental pathways, and genes [40]. A large body of work dissecting the conserved developmental and genetic basis of flower development—i.e., the floral bauplan—provides us with an excellent opportunity for exploring origins of novel organs [12, 41, 42]. Here, we provide evidence supporting that, similar to coronas in daffodils and passionflowers, the *J. calliantha* corona emerged from the novel recruitment of the stamen identity program. However, variation in the position, specific morphology, and function of these independently derived coronas is probably the result of mixed developmental origins and the differential regulation of specific downstream genes controlled by corona-specific ABCDE protein tetramer complexes.

### Evidence for a predominantly staminal origin of the *J. calliantha* corona

As is the case for many angiosperm coronas [42, 43], development of the *J. calliantha* corona is not apparent until late stages of floral ontogeny, after the establishment of the four conventional floral organ types [32]. Thus, this type of corona could be alternately interpreted as an elaboration of preexisting floral organs (as in Caryophyllaceae and Dilleniaceae) [8, 44], or as a novel organ developing from the receptacle (e.g., daffodils and passionflowers) either through neoheterochronic reiteration (sensu [45]) of a single floral organ type or as a late-stage developmental novelty with only partial homology to one or more floral organs [45, 46]. Support for the former hypothesis comes from several lines of evidence, including positional developmental information, cell micromorphology, and patterns of MADS-box gene expression, as well as intermediate morphology in flowers of hybrids between *Jaltomata* species with and without a corona (Additional file 1: Fig. S1b,c) (J. L. Kostyun, unpub.). Such hybrid flowers did not present coronas; however, they did have large expanded stamen bases that produced small openings between the ovary and the abaxial side of the stamen bases in which nectar pooled—the same position as corona wells in *J. calliantha*.

*Jaltomata calliantha* corona initials first emerge from the abaxial base of stamens, after which they merge through both lateral and medial elongation along the receptacle floor. Such a staminal origin for the corona is supported by the similarity in epidermal cell shapes between the stamen base and the corona. However, a similar cell type is also found on the adaxial surface of petals, suggesting that the stamen base, which is distinct from the stamen filament as well as anther locules, might have been partially transformed in identity before or concomitant with the origin of the corona. The observation that both petals and coronas have small rapidly dividing internal cells that are differentially stained with toluidine blue, provides some evidence for partial transformation of staminode coronas to somewhat petal-like structures. Further work using micro-CT scanning to test for shared vasculature between organs could provide another avenue for testing homologies between coronas, stamens, and/or petals [47].

Expression of BCE genes in the *J. calliantha* corona is further consistent with its having a predominantly staminal origin. Both *JcPI/GLO* orthologs (*JcGLO1* and *JcGLO2*) as well as *JcAP3* are expressed as typical B-class genes in petals and stamens, and within the corona, whereas *JcAGL1* C-class gene transcripts are confined to stamens, carpels, and the corona. An interesting observation was that *JcAG* does not show the expected expression pattern in stamens and carpels, which is in contrast to its close ortholog *pMADS3* in petunia [14, 18], and *TAG1* in tomato (*Solanum lycopersicum*) (Solanaceae) [20, 48]. Rather, *JcAG* is expressed in leaves, sepals, and petals, and at relatively low levels in coronas. The presence of a premature stop codon within a K-domain insertion might explain this change in expression, and combined with its low expression sheds doubt on its influence on corona morphology. It will, however, be interesting to determine in the future whether *JcAG* and *JcAP1* expression in coronas, but not stamens, affects the floral quartet, and thus the precise identity of the resulting structure. This will require assessment of protein–protein interactions (e.g., by yeast-two-hybrid assays), and gene-silencing or editing approaches in *Jaltomata*.

### Parallelism in the convergent evolution of floral coronas

The expression of ABCDE genes in coronas has previously been determined in a couple of distantly related species, wherein the coronas either emerge as composite structures from the tepals, hypanthium, and androgynophore (*Passiflora caerulea*) [10, 49] or from the hypanthium alone (*Narcissus bulbocodium*) [7]. In *P. caerulea*, the mixed identity of distinct corona parts is reflected by differences in B- and C-class genes. Perhaps surprisingly, the outer radii and palii, and inner operculum and annulus that develop from the tepals and hypanthium express B- and C-class genes, similar to the stamens, whereas the limen that emerges from the androgynophore only expresses the B-class gene relative *TM6*. Homology based on *AP1*-like A-class gene expression is somewhat equivocal, with expression throughout all floral organs (including the corona) during early organogenesis, but suggesting a tentative link between the corona and perianth based on late-stage expression [49]. Taken together, the relative position (*P. caerulea*) and apparent petaloidy (both species) are not necessarily predictive of the underlying genes involved.

A potential basis for the utilization of a stamen identity program, as observed in the coronas of daffodils, passionflowers, and *J. calliantha*, is consistent with its flexibility in making different types of structures with varied functions [9]. For example, staminodia, which represent clear cases of stamen transformation either inside, outside, or in place of the stamen whorl, are widespread within angiosperms, but in the cases tested, still express B- and C- class genes [7]. In *Aquilegia vulgaris* (columbine, Ranunculaceae), the molecular model for transformation of fertile stamens to staminodia is the sub- and/or neo-functionalization of duplicated *AP3/DEF* genes that are differentially expressed in late-stage stamen and staminodia development [7, 50]. A similar mechanism for diversification of staminodia has been invoked based on the duplication and differential loss of *PI/GLO* genes in Zingiberales [51]. Differential expression of the *J. calliantha AG* genes *JcAG* and *JcAGL1* might be implicated in morphological differences between coronas and stamens. Testing the generality of subtle changes in the highly conserved, but often redundant (and therefore robust), stamen identity program to affect morphological novelty will require further sampling of diverse taxa, and the use of transgenic tools to better determine gene function.

## Additional files

**Additional file 1.Fig. S1. Variation in** ***Jaltomata*** **late-stage flower morphology.** (a) *J. repandidentata* flowers produce clear nectar but do not have expanded stamen bases or coronas. (b-c) F1 hybrids between *J. repandidentata* and *J. calliantha* tend to have the blood red nectar and partially swollen stamen bases of *J. calliantha*, but do not produce coronas. This suggests that the swollen stamen base and corona development are genetically and/or developmentally associated, but can be genetically unlinked. (d) *J. sinuosa* flowers have a deep purple stamen base that is expanded laterally, but lack a corona.

**Additional file 2.Fig. S2. Quantitative RT-PCR of B and E genes showing general expression across** ***J. calliantha*** **floral organs and development.** (a) The *PI/GLO* B-class gene *JcGLO1* is expressed as predicted in petals and stamens and is also transcribed in the corona. (b) The *AP3/TM6* B-class gene *JcTM6* is expressed in both leaves and all floral organs. (c) The E-class gene *JcTM29* is specific to all floral organs. Bars in graphs denote averages of three biological replicates with standard errors. Colors mark expression in the same floral organs between the graphs and floral diagrams. Leaves, black; sepals, green; petals, blue; stamens, yellow; coronas, red; carpels, orange.

## References

[1] Ruelens P, Zhang Z, van Mourik H, Maere S, Kaufmann K, Geuten K (2017). The origin of floral organ identity quartets. Plant Cell..

[2] Stewart TA, Bhat R, Newman SA (2017). The evolutionary origin of digit patterning. EvoDevo.

[3] Harrison CJ, Morris JL (2018). The origin and early evolution of vascular plant shoots and leaves. Philos Trans R Soc Lond B Biol Sci.

[4] Abouheif E (1997). Developmental genetics and homology: a hierarchical approach. Trends Ecol Evol.

[5] Oakley TH (2017). Furcation and fusion: the phylogenetics of evolutionary novelty. Dev Biol.

[6] Martin KJ, Rasch LJ, Cooper RL, Metscher BD, Johanson Z, Fraser GJ (2016). Sox2 + progenitors in sharks link taste development with the evolution of regenerative teeth from denticles. Proc Natl Acad Sci USA.

[7] Waters MT, Tiley AMM, Kramer EM, Meerow AW, Langdale JA, Scotland RW (2013). The corona of the daffodil *Narcissus bulbocodium* shares stamen-like identity and is distinct from the orthodox floral whorls. Plant J.

[8] Endress PK, Matthews ML (2006). Elaborate petals and staminodes in eudicots: diversity, function, and evolution. Org Divers Evol.

[9] Ronse De Craene LP, Brockington SF (2013). Origin and evolution of petals in angiosperms. Plant Ecol Evol.

[10] Hemingway CA, Christensen AR, Malcomber ST (2001). B- and C- class gene expression during corona development of the blue passionflower (*Passiflora caerulea*, Passifloraceae). Am J Bot.

[11] Litt A (2007). An evaluation of a function: evidence from the *APETALA1* and *APETALA2* gene lineages. Int J Plant Sci.

[12] Coen ES, Meyerowitz EM (1991). The war of the whorls: genetic interactions controlling flower development. Nature.

[13] Rijpkema A, Gerats T, Vandenbussche M (2006). Genetics of floral development in *Petunia*. Adv Bot Res.

[14] Heijmans K, Ament K, Rijpkema AS, Zethof J, Wolters-Arts M, Gerats T, Vandenbussche M (2012). Refining the C and D in the petunia ABC. Plant Cell.

[15] Litt A, Irish VF (2003). Duplication and diversification in the *APETALA1/FRUITFULL* floral homeotic lineage: implications for the evolution of floral development. Genetics.

[16] Vrebalov J, Ruezinsky D, Padmanabhan V, White R, Medrano D, Drake R, Schuch W, Giovannoni J (2002). A MADS-box gene necessary for fruit ripening at the tomato ripening-inhibitor (rin) locus. Science.

[17] Vandenbussche M, Zethof J, Royaert S, Weterings K, Gerats T (2004). The duplicated B-class heterodimer model: whorl-specific effects and complex genetic interactions in *Petunia hybrida* flower development. Plant Cell.

[18] Kapoor M, Tsuda S, Tanaka Y, Mayama T, Okuyama Y, Tshuchimoto S, Takatsuji H (2002). Role of petunia pMADS3 in determination of floral organ and meristem identity, as revealed by its loss of function. Plant J.

[19] Quinet M, Bataille G, Dobrev PI, Capel C, Gomez P, Capel J, Lutts S, Motyka V, Angosto T, Lozano R (2014). Transcriptional and hormonal regulation of petal and stamen development by *STAMENLESS*, the tomato (*Solanum lycopersicum* L.) orthologue to the B-class *APETALA3* gene. J Exp Bot.

[20] Gimenez E, Castaneda L, Pineda B, Pan I, Moreno V, Angosto T, Lozano R (2016). *TOMATO AGAMOUS1* and *ARLEQUIN/TOMATO AGAMOUS*-*LIKE1* MADS-box genes have redundant and divergent functions required for tomato reproductive development. Plant Mol Biol.

[21] Busi MV, Bustamante C, D’Angelo C, Hidalgo-Cuevas M, Boggio SB, Valle E, Zabaleta E (2003). MADS-box genes expressed during tomato seed and fruit development. Plant Mol Biol.

[22] Angenent GC, Franken J, Busscher M, van Dijken A, van Went JL, Dons HJM, van Tunen AJ (1995). A novel class of MADS box genes is involved in ovule development in petunia. Plant Cell.

[23] Pinyopich A, Ditta GS, Savidge B, Liljegren SJ, Baumann E, Wisman E, Yanofsky MF (2003). Assessing the redundancy of MADS-box genes during carpel and ovule development. Nature.

[24] Theissen G, Melzer R, Rumpler F (2016). MADS-domain transcription factors and the floral quartet model of flower development: linking plant development and evolution. Development..

[25] Pelaz S, Ditta GS, Baumann E, Wisman E, Yanofsky MF (2000). B and C floral organ identity functions require *SEPALLATA* MADS-box genes. Nature.

[26] Ditta G, Pinyopich A, Robles P, Pelaz S, Yanofsky MF (2004). The *SEP4* gene of *Arabidopsis thaliana* functions in floral organ and meristem identity. Curr Biol.

[27] Ferrario S, Immink RG, Shchennikova A, Busscher-Lange J, Angenent GC (2003). The MADS box gene *FBP2* is required for *SEPALLATA* function in petunia. Plant Cell..

[28] Leiva González S, Mione T, Yacher L, Silvestre VQ (2010). *Modillonia* una nueva sección de *Jaltomata* Schlechtendal (Solanaceae) con una nueva especies del Norte del Perú. Arnaldoa..

[29] Wu M, Kostyun JL, Hahn MW, Moyle LC (2018). Dissecting the basis of novel trait evolution in a radiation with widespread phylogenetic discordance. Mol Ecol.

[30] Leiva González S, Mione T, Yacher L (2016). Las especies de Jaltomata Schltdl. (Solanaceae) con nectar rojo, tres nuevas combinaciones nomenclaturales y un nuevo taxón del noreste de Perú. Arnaldoa.

[31] Plourd KC, Mione T (2016). Pollination does not affect floral nectar production, and is required for fruit-set by a hummingbird-visited Andean plant species. Phytologia..

[32] Kostyun JL, Preston JC, Moyle LC (2017). Heterochronic developmental shifts underlie floral diversity within *Jaltomata* (Solanaceae). EvoDevo..

[33] Becker A, Theissen G (2003). The major clades of MADS-box genes and their role in the development and evolution of flowering plants. Mol Phylog Evol..

[34] Katoh K, Standley DM (2013). MAFFT multiple sequence alignment software version 7: improvements in performance and usability. Mol Biol Evol.

[35] Maddison WP, Maddison DR. Mesquite: a modular system for evolutionary analysis. 2018; Version 3.5.1. http://www.mesquiteproject.org. Accessed 3 Jan 2019.

[36] Huelsenbeck JP, Ronquist F (2001). MRBAYES: bayesian inference of phylogeny. Bioinformatics.

[37] Miller MA, Pfeiffer W, Schwartz T. Creating the CIPRES science gateway for inference of large phylogenetic trees. In: Proceedings of the gateway environments workshop (GCE), New Orleans, LA; 2010. pp. 1–8.

[38] Untergasser A, Cutcutache I, Koressaar T, Ye J, Faircloth BC, Remm M, Rozen SG (2012). Primer3—new capabilities and interfaces. Nucleic Acids Res.

[39] Mizukami Y, Huang H, Tudor M, Hu Y, Ma H (1996). Functional domains of the floral regulator AGAMOUS: characterization of the DNA binding domain and analysis of dominant negative mutations. Plant Cell..

[40] Brakefield PM (2011). Evo-devo and accounting for Darwin’s endless forms. Philos Trans R Soc Lond B Biol Sci..

[41] Kramer EM, Holappa L, Gould B, Jaramillo MA, Setnikov D, Santiago PM (2007). Elaboration of B gene function to include the identity of novel floral organs in the lower eudicot *Aquilegia*. Plant Cell..

[42] Chanderbali AS, Berger BA, Howarth DG, Soltis PS, Soltis DE (2016). Evolving ideas on the origin and evolution of flowers: new perspectives in the genomic era. Genetics.

[43] Sajo MG, Mello-Silva R, Rudall PJ (2010). Homologies of floral structures in Velloziaceae, with particular reference to the corona. Int J Plant Sci.

[44] Craven LA (1992). Dunlop CR A taxonomic revision of Pachynema (Dilleniaceae). Aust Syst Biol..

[45] Baum D, Donoghue MJ, Cronk QCB, Bateman RM, Hawkins JA (2002). Transference of function, heterotopy and the evolution of plant development. Developmental genetics and plant evolution.

[46] Rudall PJ (2010). All in a spin: centrifugal organ formation and floral patterning. Curr Opin Plant Biol.

[47] Dirks-Mulder A, Butot R, van Schaik P, Wijnands JWPM, van den Berg R, Krol L, Doebar S, van Kooperen K, de Boer H, Kramer EM, Smets EF, Vos RA, Vrijdaghs A, Gravendeel B (2017). Exploring the evolutionary origin of floral organs of *Erycina pusilla*, an emerging orchid model system. BMC Evol Biol.

[48] Pnueli L, Hareven D, Rounsley SD, Yanofsky MF, Lifschitz E (1994). Isolation of the tomato *AGAMOUS* gene *TAG1* and analysis of its homeotic role in transgenic plants. Plant Cell..

[49] Scorza LCT, Hernandes-Lopes J, Melo-de-Pinna GFA, Dornelas MC (2017). Expression patterns of *Passiflora edulis APETALA1/FRUITFULL* homologues shed light onto tendril and corona identities. EvoDevo.

[50] Sharma B, Kramer E (2013). Sub- and neo-functionalization of *APETALA3* paralogs have contributed to the evolution of novel floral organ identity in *Aquilegia* (columbine, Ranunculaceae). New Phytol.

[51] Piñeyro-Nelson A, Almeida AM, Sass C, Iles WJ, Specht CD (2017). Change of fate and staminodial laminarity as potential agents of flora diversification in the Zingiberales. J Exp Zool B Mol Dev Evol.

