# Supplementary figures and images for: Evidence of a largely staminal origin for the Jaltomata calliantha (Solanaceae) floral corona
Source: EvoDevo. 2019 Apr 19;10:9. doi: 10.1186/s13227-019-0122-9 (PMC6475103; doi:10.1186/s13227-019-0122-9)

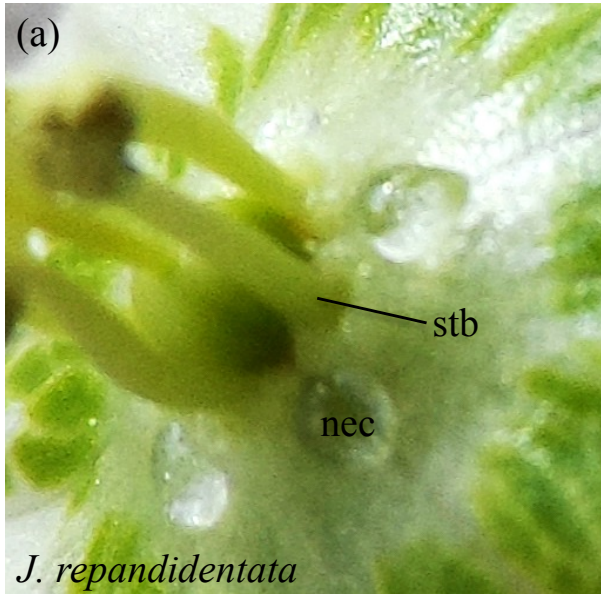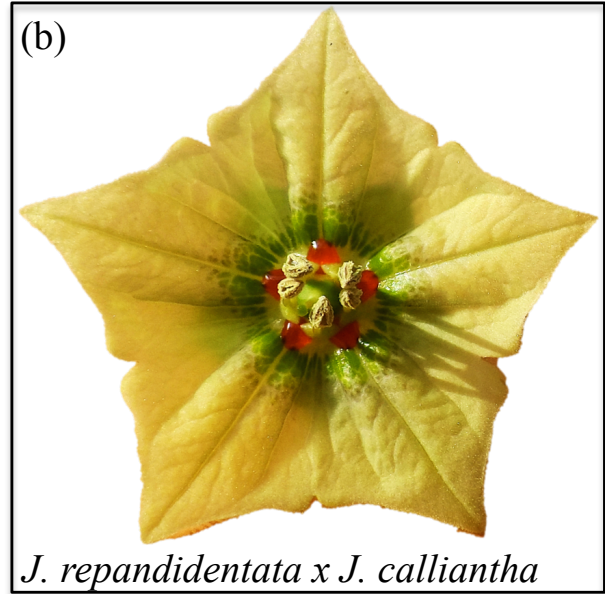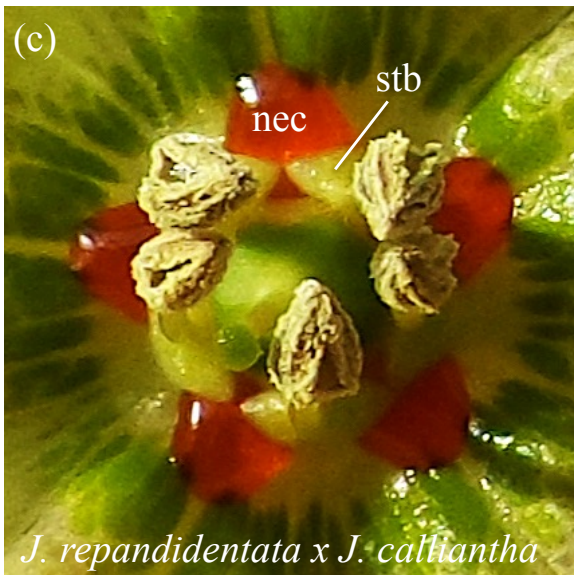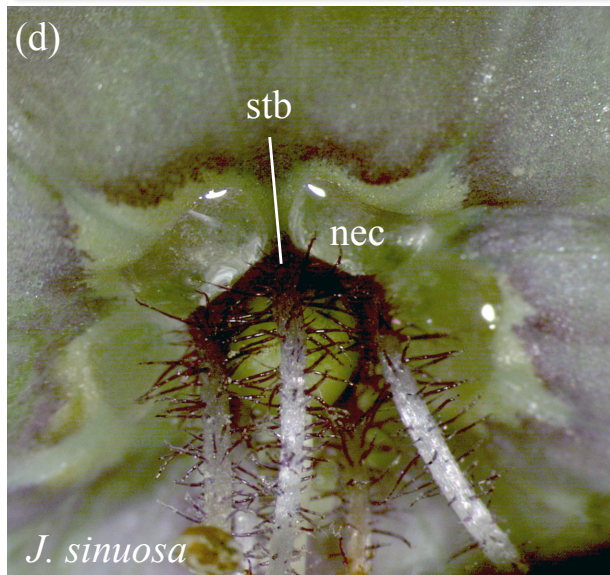

Supplement: Supplementary file 1 — Additional file 1.Fig. S1. Variation in Jaltomata late-stage flower morphology. (a) J. repandidentata flowers produce clear nectar but do not have expanded stamen bases or coronas. (b-c) F1 hybrids between J. repandidentata and J. calliantha tend to have the blood red nectar and partially swollen stamen bases of J. calliantha, but do not produce coronas. This suggests that the swollen stamen base and corona development are genetically and/or developmentally associated, but can be genetically unlinked. (d) J. sinuosa flowers have a deep purple stamen base that is expanded laterally, but lack a corona. [file 13227_2019_122_MOESM1_ESM.pdf]

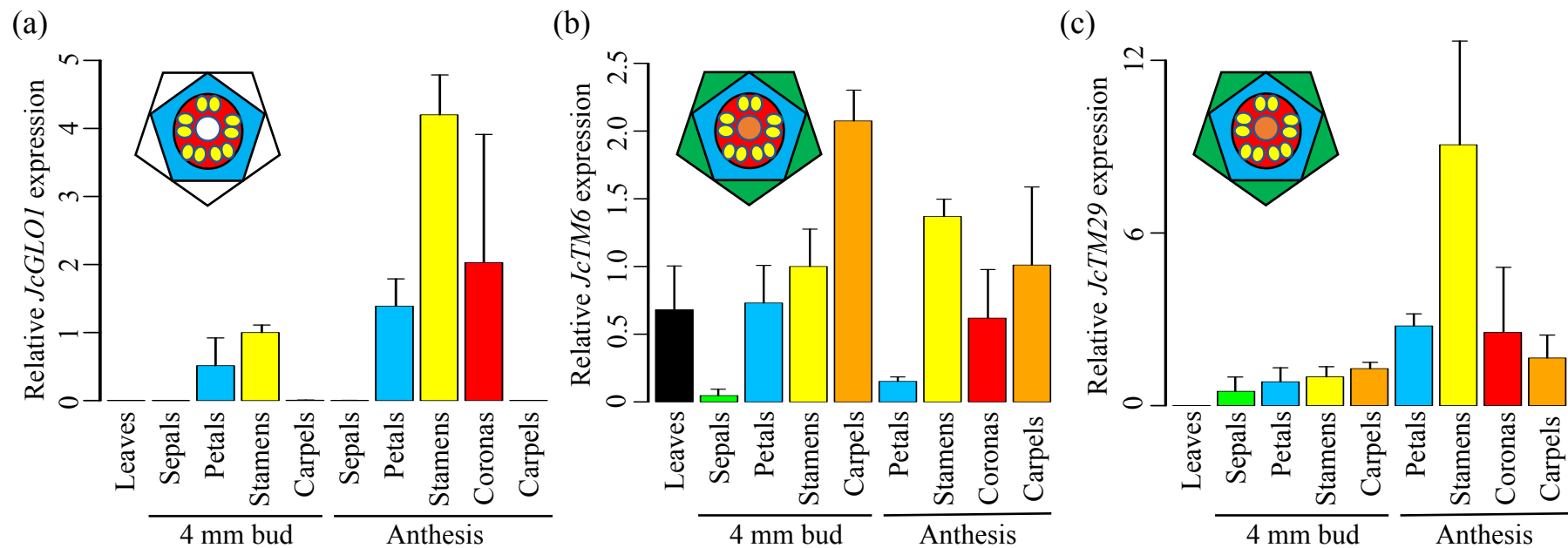

Supplement: Supplementary file 2 — Additional file 2.Fig. S2. Quantitative RT-PCR of B and E genes showing general expression across J. calliantha floral organs and development. (a) The PI/GLO B-class gene JcGLO1 is expressed as predicted in petals and stamens and is also transcribed in the corona. (b) The AP3/TM6 B-class gene JcTM6 is expressed in both leaves and all floral organs. (c) The E-class gene JcTM29 is specific to all floral organs. Bars in graphs denote averages of three biological replicates with standard errors. Colors mark expression in the same floral organs between the graphs and floral diagrams. Leaves, black; sepals, green; petals, blue; stamens, yellow; coronas, red; carpels, orange. [file 13227_2019_122_MOESM2_ESM.pdf]
